# Serum Phthalate Concentrations and Biomarkers of Oxidative Stress in Adipose Tissue in a Spanish Adult Cohort

**DOI:** 10.1021/acs.est.3c07150

**Published:** 2024-04-23

**Authors:** Celia Pérez-Díaz, Francisco M. Pérez-Carrascosa, Blanca Riquelme-Gallego, Elena Villegas-Arana, Alejandro Joaquín Armendariz, Javier Galindo-Ángel, Hanne Frederiksen, Josefa León, Pilar Requena, Juan Pedro Arrebola

**Affiliations:** † Department of Preventive Medicine and Public Health, Pharmacy School, Universidad de Granada, Campus de Cartuja s/n, 18071 Granada, Spain; ‡ Instituto de Investigación Biosanitaria (ibs.GRANADA), Avda. de Madrid, 15. Pabellón de Consultas Externas 2, 2a Planta, 18012 Granada, Spain; § Department of Nursing, Faculty of Health Sciences, C/ Cortadura del Valle Sn, 51001 Ceuta, Spain; ∥ Department of Growth and Reproduction, Copenhagen University Hospital, Rigshospitalet, Blegdamsvej 9, 2100 Copenhagen, Denmark; ⊥ International Center for Research and Research Training in Endocrine Disruption of Male Reproduction and Child Health (EDMaRC), Copenhagen University Hospital, Rigshospitalet, Blegdamsvej 9, 2100 Copenhagen, Denmark; # CIBER en Enfermedades Hepáticas y Digestivas (CIBEREHD), Av. Monforte de Lemos, 3-5. Pabellón 11. Planta 0, 28029 Madrid, Spain; ∇ Unidad de Gestión Clínica de Aparato Digestivo, Hospital Universitario San Cecilio de Granada, Av. del Conocimiento, s/n, 18016 Granada, Spain; ○ Consortium for Biomedical Research in Epidemiology and Public Health (CIBERESP), Instituto de Salud Carlos III, C/ Monforte de Lemos 3-5, Pabellón 11. Planta 0, 28029 Madrid, Spain

**Keywords:** phthalates, oxidative stress, cohort, serum, adipose tissue

## Abstract

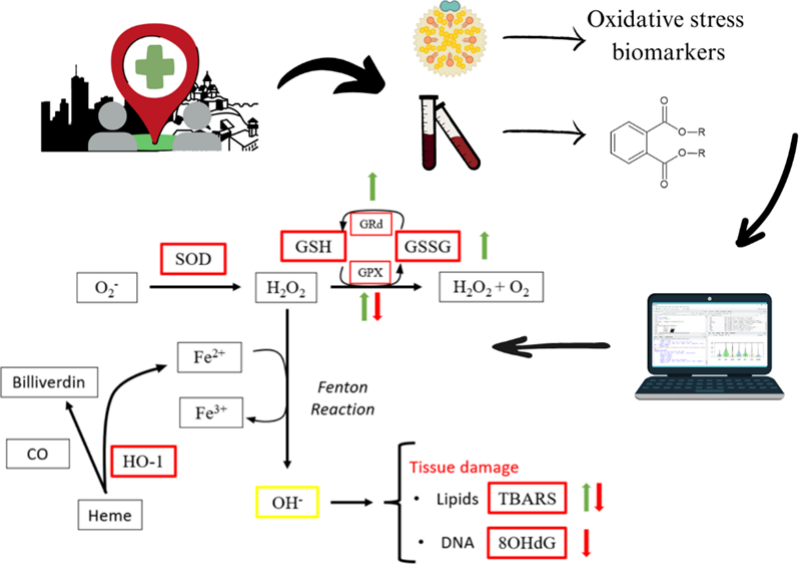

The relationship between phthalates, a group of chemical pollutants classified as endocrine disruptors, and oxidative stress is not fully understood. The aim of the present hospital-based study was to explore the associations between circulating levels of 10 phthalate metabolites and 8 biomarkers of oxidative stress in adipose tissue. The study population (*n* = 143) was recruited in two hospitals in the province of Granada (Spain). Phthalate metabolite concentrations were analyzed by isotope diluted online-TurboFlow-LC–MS/MS in serum samples, while oxidative stress markers were measured by commercially available kits in adipose tissue collected during routine surgery. Statistical analyses were performed by MM estimators’ robust linear regression and weighted quantile sum regression. Mainly, positive associations were observed of monomethyl phthalate (MMP), monoiso-butyl phthalate (MiBP), and mono-*n*-butyl phthalate (MnBP) (all low molecular weight phthalates) with glutathione peroxidase (GPx) and thiobarbituric acid reactive substances (TBARS), while an inverse association was found between monoiso-nonyl phthalate (MiNP) (high molecular weight phthalate) and the same biomarkers. WQS analyses showed significant effects of the phthalate mixture on GSH (β = −30.089; *p*-value = 0.025) and GSSG levels (β = −19.591; *p*-value = 0.030). Despite the limitations inherent to the cross-sectional design, our novel study underlines the potential influence of phthalate exposure on redox homeostasis, which warrants confirmation in further research.

## Abstract 2

This study shows novel associations between phthalate exposure and oxidative stress biomarkers in adipose tissue. This is relevant since most humans are exposed to phthalates daily, and oxidative stress is linked to several chronic diseases.

## 1 Introduction

Phthalates are widely used as additives in plastic manufacturing of different products such as toys, cosmetics, or food packaging.^1−3^ Phthalates are frequently divided into two main groups: high molecular weight phthalates (HMWP) and low molecular weight phthalates (LMWP). The former are mainly used as plasticizers in various plastic products, while the latter are commonly used as additives in cosmetics or medicines among others.^4^ Despite various restrictive policies^5^ and some voluntary changes in the market,^6^ phthalate production remains high. In 2018, 5.5 million tonnes were produced.^7^ In addition, despite their short half-life (about 24–48 h),^8−10^ the population is constantly exposed to phthalates due to their ubiquity in daily use products.^6^ Moreover, phthalates are moderately lipophilic and can diffuse into the lipid bilayer of cells and spread to different tissues via the cardiovascular system.^11,12^ Therefore, this sustained and spread exposure explains the associations found in previous studies between phthalates and/or their metabolites and different health conditions, including metabolic syndrome, diabetes, dyslipidemia, or cancer.^13−19^

Oxidative stress is known as an imbalance in the production and detoxification of reactive oxygen species (ROS)^20^ and reactive nitrogen species (RNS).^21−23^ Several physiological processes such as activation of various transcriptional factors, apoptosis, immunity, protein phosphorylation, and differentiation depend on the proper production and presence of these radicals inside cells since ROS are capable of causing damage at the cellular level^22,24^ through reaction with susceptible compounds such as lipids, proteins and/or DNA.^22,24,25^ There are different molecular mechanisms to prevent this damage, including the redox cycle, in which the superoxide anion radical (O^2–^), considered a primary ROS, is eventually neutralized and transformed into water and oxygen.^26^ However, in cases of system dysfunction, the Fenton reaction may be induced, resulting in the transformation of the intermediate hydrogen peroxide (H_2_O_2_) into the hydroxyl radical (HO^–^). This transformation leads to tissue damage at macromolecular levels, e.g., lipid oxidation or DNA alterations.^22,26^

The main endogenous source of ROS is the mitochondrion along the electron transport chain. Exogenous ROS sources include lifestyle patterns, e.g., lack or excess of physical exercise, consumption of certain foods, especially those high in fats and sugars, stress, cigarette smoke, and some drugs such as anesthetics and chemotherapeutics.^26−30^ In addition, inadvertent long-term exposure to certain chemical environmental pollutants (e.g., bisphenols, parabens, or chromium) is acknowledged to contribute to the oxidative unbalance.^31−33^ Moreover, there have been recent concerns about the potential contribution of phthalate exposure to redox-related chronic diseases, e.g., cancer or diabetes.^3,34,35^ However, the relationship between phthalate exposure and oxidative stress remains unclear.

Several human and animal studies have underlined the relevance of adipose tissue disruption in the onset of a number of noncommunicable chronic diseases (e.g., cardiovascular disease or diabetes). Particularly, in situ redox unbalance might have systemic implications and, therefore, promote the development of these prevalent conditions.^1,36−41^

The present study aims to shed light on the potential effect of phthalate metabolites on metabolic health by investigating the associations of serum phthalate metabolite concentrations and oxidative stress biomarkers in adipose tissue from a Spanish adult cohort.

## 2 Materials and Methods

### 2.1 Study Population: GraMo Cohort

The present study is based in a subsample of the GraMo cohort, previously characterized elsewhere.^42−45^ In brief, participants were recruited between July 2003 and June 2004 among surgery patients who underwent a routine intervention unrelated to an oncological process in order to obtain an adipose tissue sample. The recruitment took place in two public hospitals: Hospital Clínico San Cecilio in Granada (inland, urban area) and Hospital Santa Ana in Motril (coastal semirural area).

**Figure 1:**
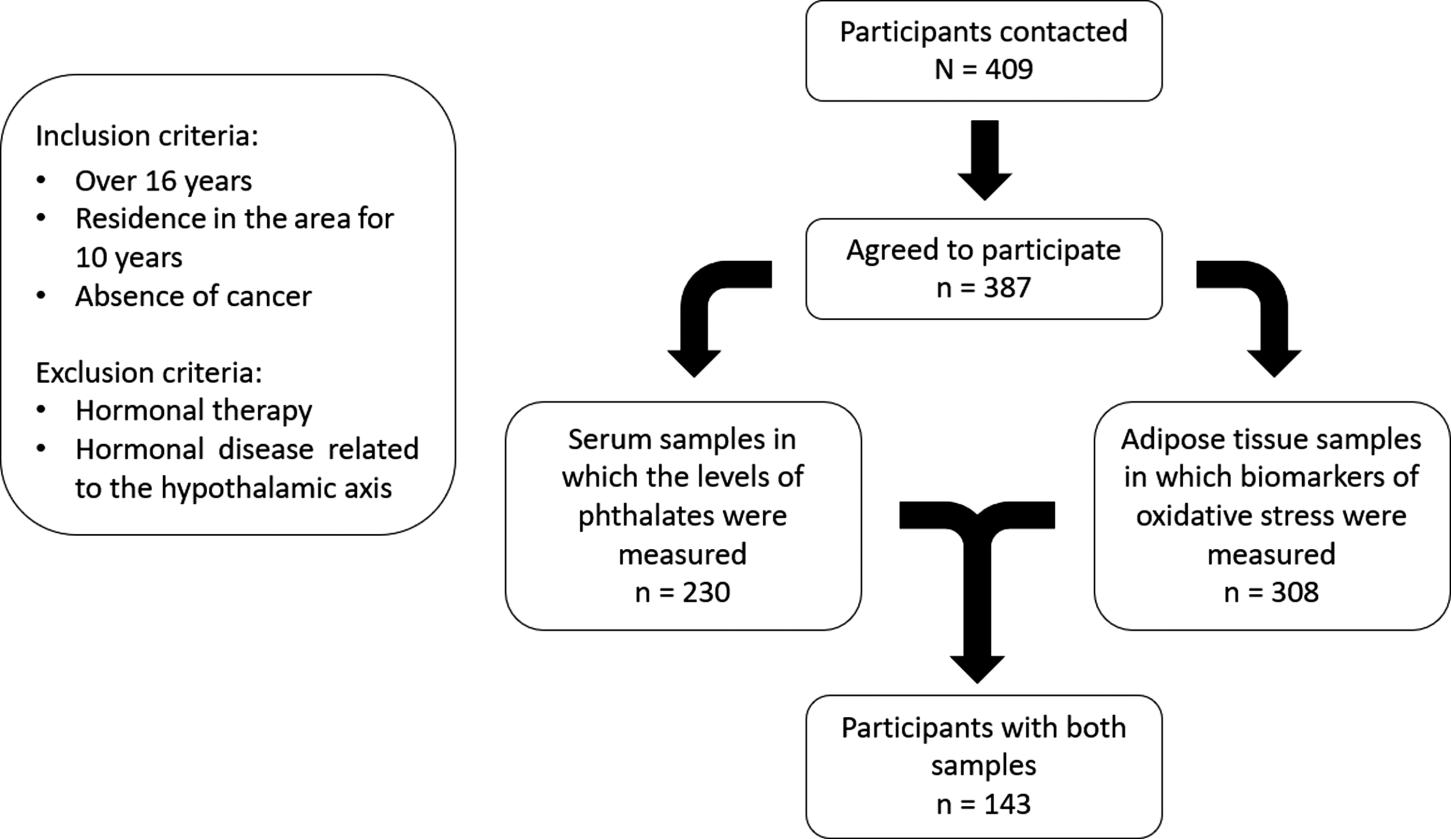
Selection criteria for GraMo cohort participants and flowchart of participants selected for the study subsample.

Out of 409 individuals meeting the selection criteria who were contacted, 405 donated 12 h fasting blood samples. Phthalate metabolites were measured in serum samples from 230 individuals, and oxidative stress biomarkers were assessed in adipose tissue samples of 308 participants. A total of 143 individuals had measures of both biomarkers (Figure 1). There were no statistically significant differences between the characteristics of participants in the subsample and those in the full cohort (data not shown).

Data on sociodemographic characteristics, lifestyle, and health status were gathered in face-to-face interviews conducted by trained personnel at the time of recruitment during hospitalization. Body mass index (BMI) was expressed as weight/height squared (kg/m^2^) and a participant was categorized as obese with a BMI > 30. A participant was considered a smoker or alcohol consumer with any level of daily tobacco (≥1 cigarette/day) or weekly alcohol (≥1 drink/week). An individual was considered to have type 2 diabetes if he/she had ever been diagnosed with diabetes by a clinician. Additionally, medical records were reviewed, whenever a participant showing a fasting glucose level ≥ 126 mg/dL in the routine analyses prior to the surgery, he/she was registered as diabetic. No discrepancies were found between the self-reported prevalence of diabetes and the data reported in the clinical records. For hypertension (systolic blood pressure > 140 mmHg and/or diastolic blood pressure > 90 mmHg), a participant was considered hypertensive if he/she had a previous diagnosis in clinical records.

All participants signed their informed consent to participate in the study, which was approved by the Ethics Committee of each hospital in 2002 for the recruitment of patients and analysis of pollutants and by the Ethics Committee of Granada (Comité de Ética de Investigación Clnica de Granada, 8/2016) for the analysis of stress biomarkers.

### 2.2 Laboratory Analysis

#### 2.2.1 Phthalate Metabolite Assessment

The concentration of a total of 32 phthalate metabolites from 15 different phthalate diesters was measured by isotope diluted online-TurboFlow-LC–MS/MS with preceding enzymatic deconjugation. Samples were analyzed randomly. They were divided into 5 blinded batches, each including around 45 samples plus calibration standards, three blanks, three serum pool controls, and three serum pool controls spiked with phthalate metabolite standards at low or high known concentrations. The interday variation analyzed as the relative standard deviation between batches was < 21% for all analytes spiked in serum at a low level and < 11% for all analytes spiked in serum at a high level. The method used for sample preparation, calibration, and control quality control materials, as well as instrumental analysis and method validation has been described in detail previously. The method was used without any modifications.^46^

Phthalate metabolites with a detection range < 20% were excluded (Supplementary Table S1). Thus, of the 32 phthalate metabolites initially screened, only 10 were included in the present study, namely, monomethyl phthalate (MMP), monoethyl phthalate (MEP), monoiso-butyl phthalate (MiBP), mono-*n*-butyl phthalate (MnBP), mono-(2-ethyl-hexyl) phthalate (MEHP), mono-(2-ethyl-5-carboxypentyl) phthalate (MECPP), mono-(2-carboxymethyl-hexyl) phthalate (MCMHP), monoiso-nonyl phthalate (MiNP), monobenzyl phthalate (MBzP), and monoisodecyl phthalate (MiDP). In the regression models, all metabolite concentrations were treated as continuous variables, and values below the limit of detection (LOD) were replaced by random numbers between 0 and their respective LOD.^47^ The exceptions were MBzP (23.1% < LOD) and MiDP (46.2% < LOD), which were dichotomized (>LOD/<LOD).

#### 2.2.2 Oxidative Stress Biomarker Assessment

Oxidative stress biomarkers were measured in adipose tissue using commercially available kits (Enzo Life Sciences, Inc., Farmingdale, NY, USA) in an automated microplate reader (TRIAD MRX II series, Dynex Technologies Inc., Chantilly, Virginia, USA).

The adipose tissue samples were slowly thawed on ice and washed repeatedly with cold PBS to remove blood clots and other remnants. They were then homogenized in the appropriate buffer at the ratio specified by each kit using a pestle and mortar. The following biomarkers were assessed: glutathione peroxidase (GPx), glutathione reductase (GRd), total glutathione (GST), reduced glutathione (GSH), oxidized glutathione (GSSG), hemeoxygenase-1 (HO-1), superoxide dismutase (SOD) activity, thiobarbituric acid reactive substances (TBARS), in which malondialdehyde reacts with thiobarbituric acid, and 8-hydroxy-deoxyguanosine (8OHdG).

For the latter, the number of participants was restricted to 112 based on adequate biological sample availability for the analyses.

The GSH value was determined by subtracting the GSSG levels from the GST levels. The GSSG/GSH ratio was obtained by dividing the GSSG levels by the GSH levels in the same sample. Detailed methodological information of these analyses is provided elsewhere.^26,48^

### 2.3 Statistical Analysis

The descriptive analysis included the calculation of medians and interquartile ranges for continuous variables and frequencies and percentages for the categorical variables. Three groups were defined according to the tertiles of the sum of the orders of phthalate levels. In the bivariate analyses, variables were compared using the Mann–Whitney U-test and Fisher’s exact test as appropriate.

Pairwise correlations between metabolites and oxidative stress biomarkers were analyzed using the Spearman rank correlation test. Then, concentrations of each phthalate were natural log-transformed for the regression models, which relaxes the linearity assumption.

The shape of the associations between phthalate metabolite concentrations and biomarkers of oxidative stress were further explored using generalized additive models. Considering the highly skewed distribution of redox markers and contaminants, the magnitude of the associations was analyzed by means of robust regression based on the MM-estimator.^49^ The covariates included were initially selected from among those most commonly used in the available literature.^19,50,51^ Final covariate selection in the multivariable models was performed by using a combination of forward and backward stepwise methods. The model 1 was adjusted for age, sex, BMI (continuous), place of residence (urban/semirural), educational level (no studies/primary or higher), smoking (never, former, current), and alcohol consumption (regular consumer/nonconsumer) and type of work (nonmanual worker, manual worker, retired), while model 2 was adjusted by these covariates but also by the consumption of vegetable foods.

The potential mixture effect of different phthalate metabolites on oxidative stress markers was assessed by weighted-quantile-sum regression (WQS). WQS estimates a weighted index based on the combination of several exposures, considering their individual associations with the outcome. The WQS analyses of the combined effect of phthalate concentrations on oxidative stress marker levels were assessed by entering the WQS index as an independent variable in the multivariable regression with the levels of each oxidative stress marker as the dependent variable and adjusting for the same covariates as the individual associations in model 2. Considering that the WQS regression requires an a priori expected direction of the association, we estimated two mixed-effect models (positive and negative) for each outcome. All WQS analyses were performed with natural log-transformed pollutant concentrations, using a training set defined as a 30% random sample of the data set, the remaining 70% being used for model validation. The final weights were calculated by using a total of 1000 bootstrap steps.

Data were stored and processed using RStudio version 4.3.1.^52^ The following packages were used: vioplot^53^ for the creation of violin plots describing graphically the concentration of phthalate metabolites and oxidative stress biomarkers, mgcv^54^ for the creation of GAM plots and robustbase^55^ for robust regression with MM estimators. WQS analysis was performed by using gWQS.^56^

## 3 Results

**Figure 2:**
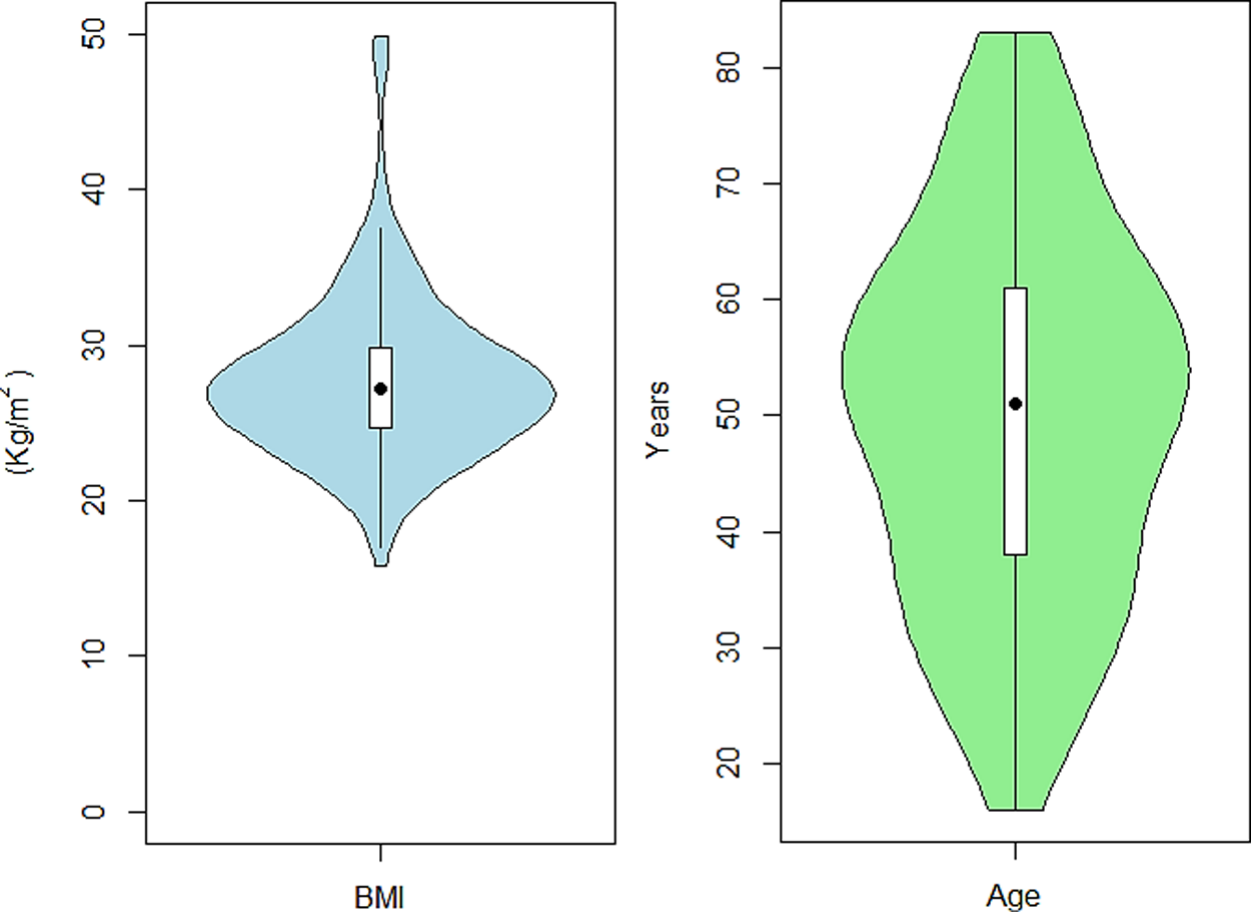
Violin plot of the distribution of participants’ BMI in kg/m^2^ and age in years, showing the median (black point) and the interquartile range (white box).

The characteristics of the study population are listed in Table 1 and are shown in Figure 2.

**Table 1:** Baseline Main Characteristics of the Subsample Adults from the GraMo Cohort According to Phthalate Metabolite Sum Levels in Tertiles (*n* = 143)

characteristics	phthalate metabolite sum			
	first tertile (lowest levels)	second tertile	third tertile (highest levels)	*p*- value
*n* [*n* (%)]	55 (38.46)	50 (34.97)	38 (26.57)	
age				0.44
median (IQR)	54 (35.5–63.0)	48.00 (35.0–58.0)	53.5 (38.0–62.3)	
missing [*n* (%)]	8 (14.6)	1 (2.0)	2 (5.3)	
BMI				0.57
median (IQR)	26.3 (23.6–30.0)	28.1 (24.3–30.1)	27.4 (25.7–29.6)	
missing [*n* (%)]	8 (14.6)	0 (0.0)	2 (5.3)	
sex [*n* (%)]				
women	22 (40.0)	24 (48.0)	17 (44.7)	
men	25 (45.5)	26 (52.0)	19 (50.0)	
missing	8 (14.5)	0 (0.0)	2 (5.3)	
hospital [*n* (%)]				<0.0001
Granada	37 (67.3)	11 (22.0)	5 (13.2)	
Motril	10 (18.2)	39 (78.0)	31 (81.6)	
missing	8 (14.5)	0 (0.0)	2 (5.3)	
education [*n* (%)]				
primary education	11 (20.0)	13 (26.0)	15 (39.5)	
secondary education or higher	36 (65.5)	37 (74.0)	20 (52.6)	
missing	8 (14.5)	0 (0.0)	3 (7.9)	
occupation [*n* (%)]				0.35
nonmanual worker	11 (20.0)	9 (18.0)	7 (18.4)	
manual worker	30 (54.5)	39 (78.0)	28 (73.7)	
retired	6 (10.9)	2 (4.0)	1 (2.6)	
missing	8 (14.5)	0 (0.0)	2 (5.3)	
surgery [*n* (%)]				0.003
hernia	21 (38.2)	22 (44.0)	19 (50.0)	
gallbladder	18 (32.7)	7 (14.0)	2 (5.3)	
varicose veins	2 (3.6)	3 (6.0)	3 (7.9)	
others	6 (10.9)	18 (36.0)	12 (31.6)	
missing	8 (14.5)	0 (0.0)	2 (5.3)	
alcohol [*n* (%)]				0.28
no consumption	24 (43.6)	22 (44.0)	12 (31.6)	
consumption	22 (40.0)	27 (54.0)	23 (60.5)	
missing	9 (16.4)	1 (2.0)	3 (7.9)	
smoke [*n* (%)]				0.93
nonsmoker	18 (32.7)	20 (40.0)	16 (42.1)	
fomer smoker	10 (18.2)	12 (24.0)	9 (23.7)	
current smoker	19 (34.5)	18 (36.0)	11 (28.9)	
missing	8 (14.5)	0 (0.0)	2 (5.3)	
legumes [*n* (%)]				0.99
never	1 (1.8)	2 (4.0)	2 (5.3)	
<1 per week	2 (3.6)	2 (4.0)	1 (2.6)	
1 per week	7 (12.7)	9 (18.0)	5 (13.2)	
twice per week	19 (34.5)	16 (32.0)	12 (31.6)	
>2 per week	18 (32.7)	20 (40.0)	15 (39.5)	
missing	8 (14.5)	1 (2.0)	3 (7.9)	
cooked vegetables [*n* (%)]				0.48
1 or < 1 per week	16 (29.1)	14 (28.0)	8 (21.1)	
twice per week	14 (25.5)	12 (24.0)	7 (18.4)	
>2 per week	17 (30.9)	23 (46.0)	20 (52.6)	
missing	8 (14.5)	1 (2.0)	3 (7.9)	
raw vegetables [*n* (%)]				0.33
never	0 (0.0)	2 (4.0)	0 (0.0)	
<1 per week	1 (1.8)	0 (0.0)	1 (2.6)	
1 per week	4 (7.3)	2 (4.0)	1 (2.6)	
twice per week	8 (14.5)	3 (6.0)	5 (13.2)	
>2 per week	34 (61.8)	42 (84.0)	28 (73.7)	
missing	8 (14.5)	1 (2.0)	3 (7.9)	
fruits [*n* (%)]				0.76
never	2 (3.6)	1 (2.0)	0 (0.0)	
<1 per week	1 (1.8)	2 (4.0)	1 (2.6)	
1 per week	3 (5.5)	0 (0.0)	1 (2.6)	
twice per week	3 (5.5)	2 (4.0)	2 (5.3)	
>2 per week	38 (69.1)	44 (88.0)	31 (81.6)	
missing	8 (14.5)	1 (2.0)	3 (7.9)	
hypertension [*n* (%)]				0.92
low	43 (78.2)	45 (90.0)	32 (84.2)	
high	3 (5.5)	5 (10.0)	3 (7.9)	
missing	9 (16.4)	0 (0.0)	3 (7.9)	
obesity [*n* (%)]				0.57
normal weight	15 (27.3)	17 (34.0)	7 (18.4)	
overweight	20 (36.4)	20 (40.0)	20 (52.6)	
obesity	12 (21.8)	13 (26.0)	9 (23.7)	
missing	8 (14.5)	0 (0.0)	2 (5.3)	
diabetes [*n* (%)]				0.44
no diabetic	46 (83.6)	48 (96.0)	32(84.2)	
diabetic	1 (1.8)	2 (4.0)	3 (7.9)	
missing	8 (14.5)	0 (0.0)	3 (7.9)	

### 3.1 Phthalates Metabolites and Oxidative Stress Biomarkers

**Figure 3:**
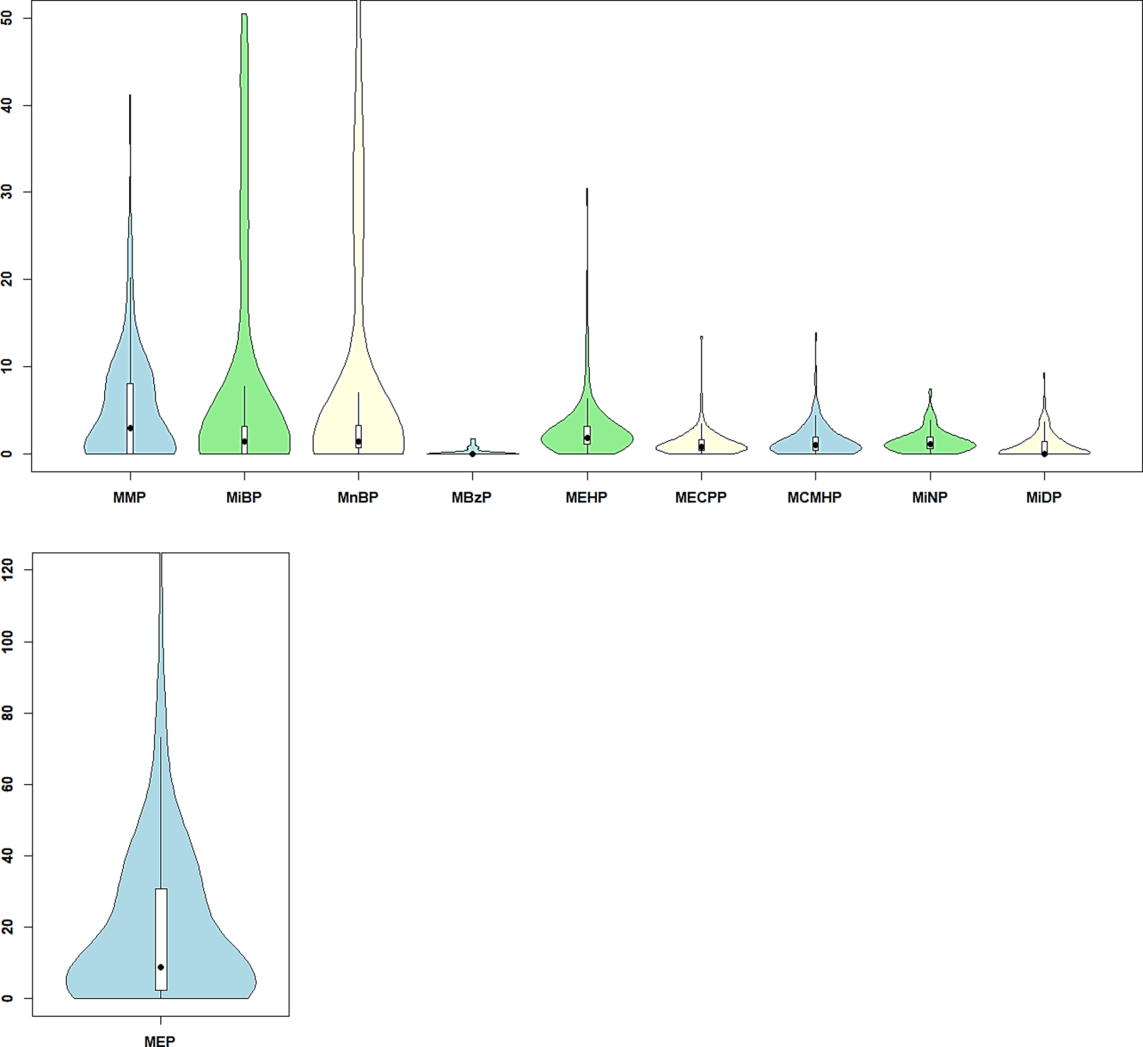
Serum concentrations of phthalate metabolites in the study population. Concentrations (ng/mL) are plotted as a violin plot, showing the median (black point) and the interquartile range (white box). MMP, monomethyl phthalate; MEP, monoethyl phthalate; MiBP, monoiso-butyl phthalate; MnBP, mono-*n*-butyl phthalate; MBzP, monobenzyl phthalate; MEHP, mono-(2-ethyl-hexyl) phthalate; MECPP, mono-(2-ethyl-5-carboxypentyl) phthalate; MCMHP mono-(2-carboxymethyl-hexyl) phthalate; MiNP, monoiso-nonyl phthalate; and MiDP, monoisodecyl phthalate.

**Figure 4:**
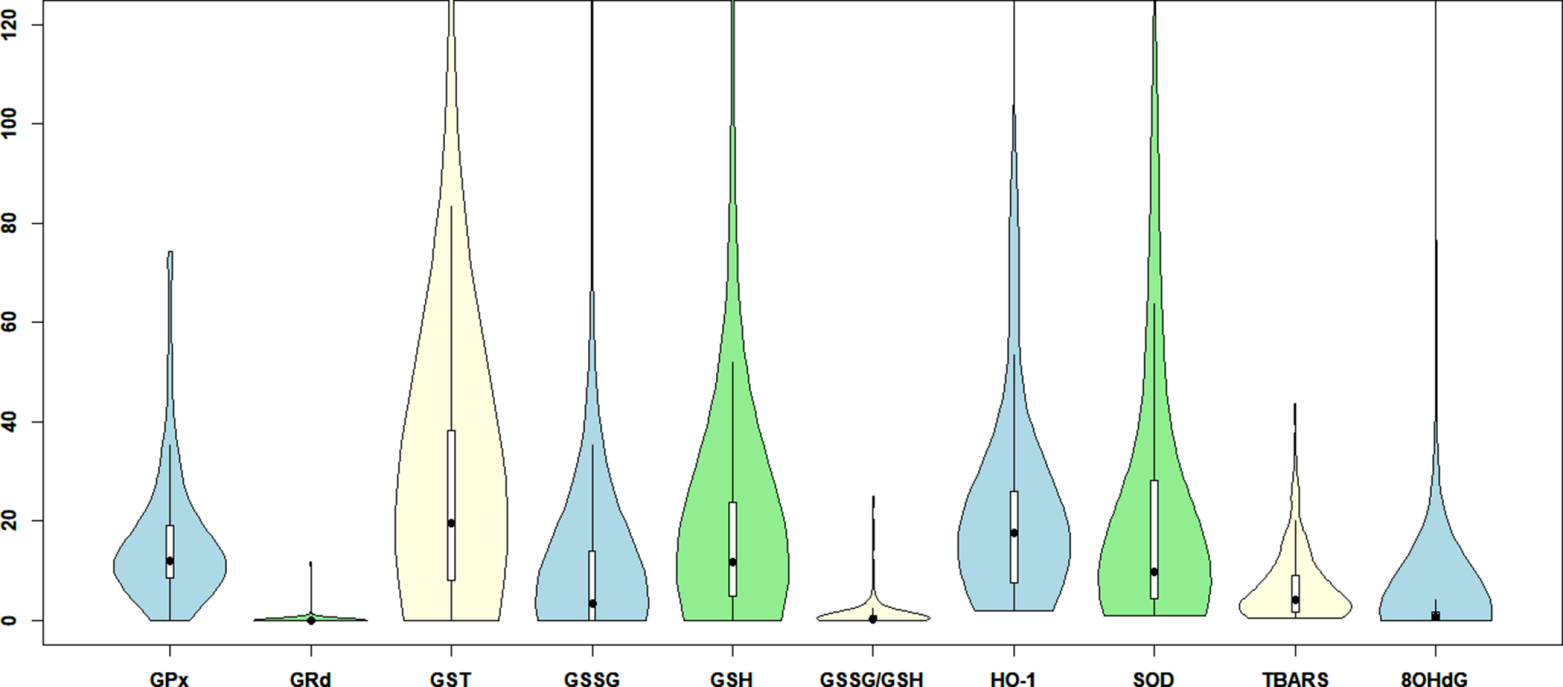
Adipose tissue concentrations of oxidative stress biomarkers in the study population. Concentrations are plotted as violin plots, showing the median (black point) and interquartile range (white box). GPx, glutathione peroxidase (nmol/min mg proteins); GRd, glutathione reductase (nmol/min mg proteins); GST, total glutathione (nmol/min mg proteins); GSH, reduced glutathione (nmol/min mg proteins); GSSG, oxidized glutathione (nmol/min mg proteins); GSSG/GSH, oxidized glutathione/reduced glutathione ratio (nmol/min mg proteins); HO-1, hemeoxygenase-1 (ng/mL) (falta las unidades en la que está la concentración); SOD, superoxide dismutase; TBARS, thiobarbituric acid reactive substances (μM/mg protein); 8OHdG, 8-hydroxy-deoxyguanosine (ng/mL).

In the present subcohort, MEP was the metabolite that was found in the highest concentrations (median, IQR), followed by MMP (Figure 3), while GST was the redox biomarker found at the highest concentrations in adipose tissue samples, followed by SOD, OH-1 and GSH (Figure 4).

The description of the levels of phthalate metabolites and oxidative stress biomarkers according to the sum of orders of phthalate metabolite levels divided in tertiles is listed in Supporting Information Table S2.

### 3.2 Association of Phthalate Metabolites with Oxidative Stress Biomarkers

**Figure 5:**
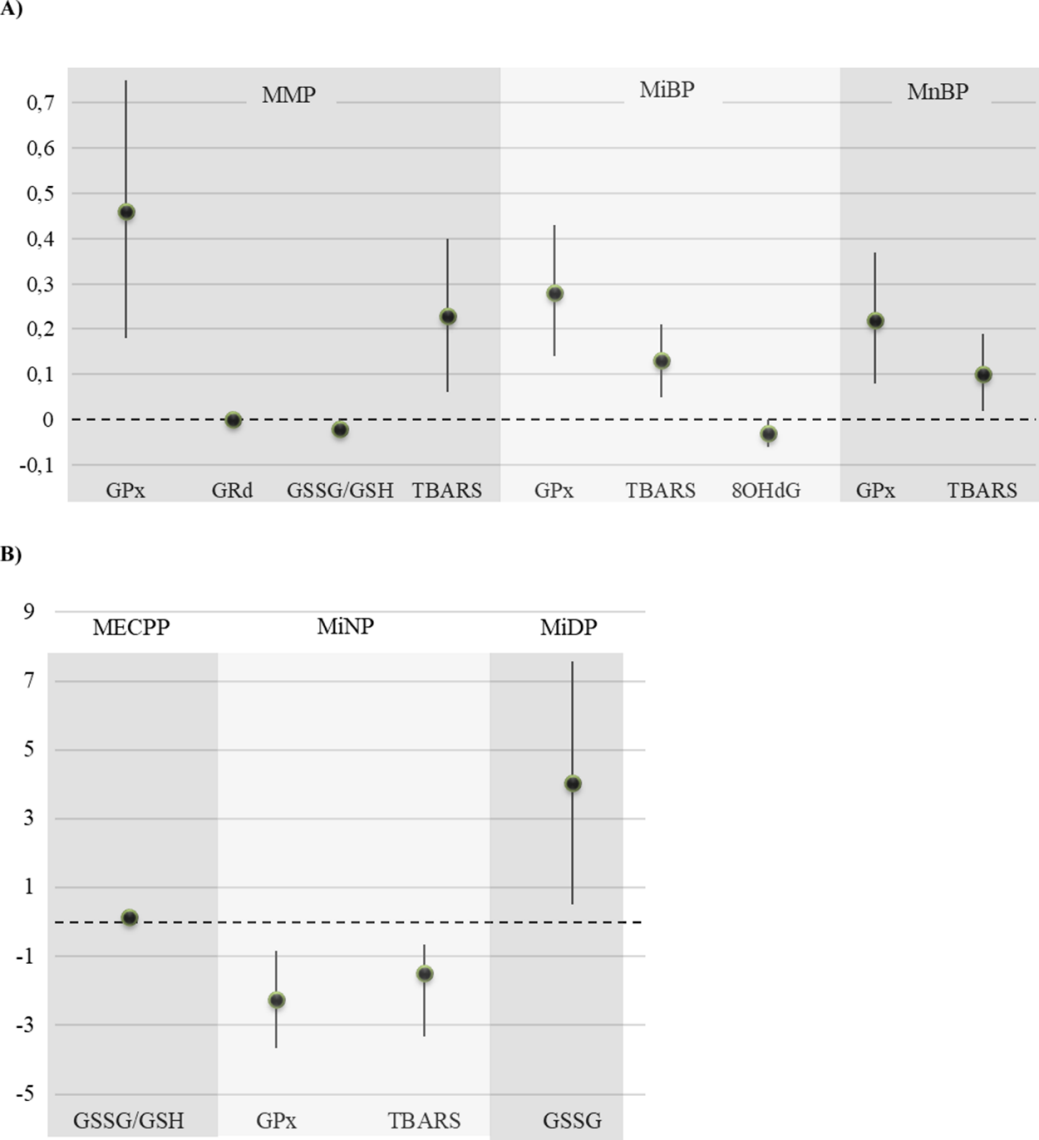
(A, B) Associations between phthalate metabolites and oxidative stress biomarkers. Adjusted regression coefficients (95% confidence intervals) for changes in natural log-transformed phthalate metabolite concentrations in relation to oxidative stress biomarkers. Adjusted for age, sex, BMI, place of residence, educational level, smoking habit, alcohol consumption, and type of work. MMP, monomethyl phthalate; MiBP, monoiso-butyl phthalate; MnBP, mono-*n*-butyl phthalate; MECPP, mono-(2-ethyl-5-carboxypentyl) phthalate; MiNP, monoiso-nonyl phthalate; MiDP, monoisodecyl phthalate. GPx, glutathione peroxidase; GRd, glutathione reductase; GSSG, oxidized glutathione; GSSG/GSH, oxidized glutathione/reduced glutathione ratio; SOD, superoxide dismutase; TBARS, thiobarbituric acid reactive substances; 8OHdG, 8-hydroxy-deoxyguanosine.

The results from multivariable robust regression models are summarized in Figure 5 and are listed in Supporting Information Table S3.

There was a positive association between several LMWP metabolites (i.e., MMP, MiBP, and MnBP) and the oxidative stress markers GPx and TBARS (Figure 5A). However, these stress markers were negatively associated with MiNP, an HMWP metabolite (Figure 5B).

Besides the mentioned pattern, MMP showed a positive association with GRd and MiDP with GSSG. In addition, MMP (inversely) and MECPP (positively) were also associated with the GSSG/GSH ratio. Regarding 8OHdG, an inverse association was observed with MiBP. However, no significant association was found between MBzP, MEP, MEHP, and MCMHP or the sum of orders of phthalate metabolites and oxidative stress markers.

**Figure 6:**
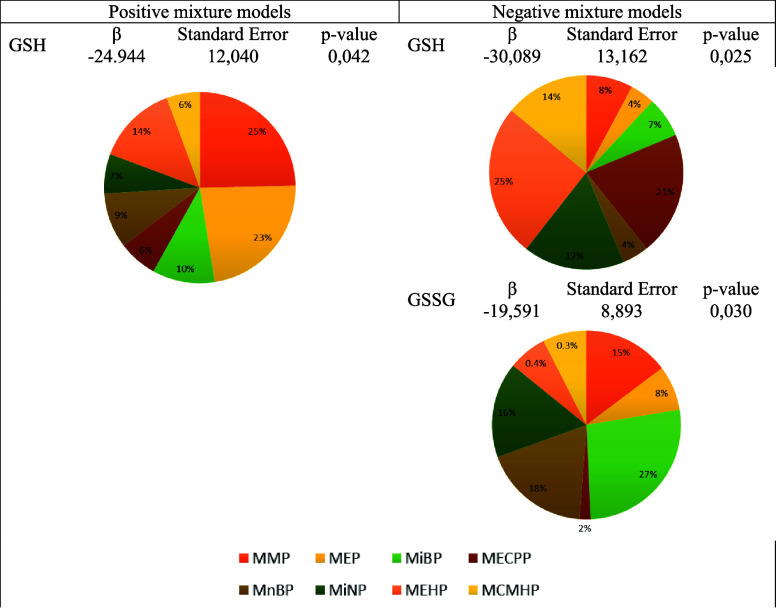
Estimation of the mixture associations of phthalate metabolites with different immuno-inflammatory biomarker levels. Weighted quantile sum regression (WQS) models. GSH, reduced glutathione (nmol/min mg proteins), GSSG, oxidized glutathione (nmol/min mg proteins); MMP, monomethyl phthalate; MEP, monoethyl phthalate; MiBP, monoiso-butyl phthalate; MECPP, mono-(2-ethyl-5-carboxypentyl) phthalate; MnBP, mono-*n*-butyl phthalate; MiNP, monoiso-nonyl phthalate; MEHP, mono-(2-ethyl-hexyl) phthalate; MCMHP mono-(2-carboxymethyl-hexyl) phthalate.

To account for the potential mixture effect of phthalate metabolites on stress biomarker levels, we calculated a WQS index as a measure of the combined effect (Supplementary Table 4). The WQS index was negatively and significantly associated with GSH levels (β = −30.089; *p*-value = 0.025,). In addition, the WQS index was negatively and significantly associated with GSSG levels (β = −19.591; *p*-value 0.030). As shown in Figure 6, in the negative mixture model of GSH, the major components were MEHP (25%) and MECPP (21%). For the negative mixture model of GSSG, the major components were MiBP (27%) and MnBP (18%).

## 4 Discussion

In the present study, we evidenced novel associations between phthalate metabolites and in situ adipose tissue redox biomarkers. Overall, GPx and TBARS were positively associated with LMWP metabolites, while their association with the HMWP metabolite MiNP was negative.

The biological plausibility of our findings is supported by results from different types of studies.

Exposure of placental cells to MEHP has been shown to increase ROS production, DNA damage, apoptosis, and altered expression of redox-sensitive genes.^57^ Moreover, another study conducted on mice follicle cells found that DEHP (the parent compound of MEHP, MECPP, and MCMHP) increased free radical levels compared to control cells. Furthermore, these levels returned to normal after the application of *n*-acetylcysteine. However, although a decrease in SOD1 was observed, no significant changes were observed in GPx and catalase.^58^ In the same direction, the in vitro study of Cho et al., 2015, found that SOD in human endometrial stromal cells was inhibited by DEHP (HMWP).^59^

Previous in vivo research in *Eisenia fetida* found enhanced SOD activity by DEP and inhibited by DBP (both LMWPs), DEHP and DOP (HMWPs).^60^ In addition, an association between the two DBP isomers; DiBP and DnBP (the parent compounds of MiBP and MnBP), and increased lipid peroxidation and other redox biomarkers were found in an in vivo model on adult zebrafish.^61^ Furthermore, in a mouse model to determine any adverse effects of DBP on allergic asthma, it was found that DBP significantly decreased GSH levels and increased 8OHdG levels.^62^ These results would be in contrast to our findings since, in our case, high levels of MiBP, a metabolite of DiBP, would be negatively related to levels of 8OHdG.

Finally, a recent in vivo study investigating the effects of a mixture of various phthalates (DEP, DEHP, DBP, DiBP, DiNP, and BzBP) on adipose tissue from female mice observed that the phthalate mixture increased GPx levels (but not SOD levels as well as our study). This association was independent of the type of adipose tissue (white or brown).^63^

In line with our findings, previous epidemiological studies also reported associations between phthalate exposure and increased oxidative stress.^24,64−68^ The systematic review by Sweeney et al.*,* 2019^69^ concluded that MiBP and MEP may be associated with some biomarkers of oxidative stress although there are some discrepancies between different works. However, to the best of our knowledge, our patterns with HMWP and LMWP metabolites have not been previously described in an epidemiological setting.

The study by Duan et al., 2017 in diabetic patients found only positive associations between phthalate metabolites and lipid peroxidation.^67^ These associations were not only observed with MMP, MiBP, and MnBP but also with other LMWP and HMWP metabolites analyzed in our cohort, except for MiNP and MiDP. The difference in results could be related to dissimilarities in the study populations, particularly considering the GraMo included both diabetics and non-diabetics. It is noteworthy that diabetes has been shown to be associated with tissue damage due to increased oxidative stress.^70^

Furthermore, our results are consistent with evidence previously found in the GraMo cohort of positive associations between phthalates and inflammatory markers such as PAI-1, MCP-1, IL-18, and leptin. It is known that when reactive oxygen species and free radicals overcome the body’s antioxidant potential, this can result in inflammation and, thus, tissue damage and/or death.^71^ It is worth mentioning that our study is one of the very first investigations focusing on redox reactions in adipose tissue, and therefore our outcome variables might have singular biological meanings. While general oxidative stress is commonly measured in serum and/or urine, in our study, we are measuring oxidative stress in a specific tissue. Thus, a low but chronic increase in adipose tissue oxidative stress may lead to an increase in the activity of the antioxidant system and, thus, to a decrease in DNA damage. This could be a potential explanation for our inverse associations with 8OHdG, a marker of oxidative DNA damage, as previous epidemiological investigations have reported positive correlations.^24,64−66^ Furthermore, the age range of the participants might also be a source of variability since other cohorts were predominantly composed of children and adolescents, while the GraMo cohort exclusively consisted of adults. This could suggest that phthalate exposure is associated with early onset of cellular DNA damage, which may not be so evident in an adult cohort, or even that exposure to phthalates modifies redox homeostasis but not enough to induce DNA damage.

When we ran the WQS regression analyses to study the mixed effect of phthalates on oxidative stress biomarkers, the associations we found in the individual analysis disappeared. However, we found other statistically and negatively significant associations between the mixture of phthalates and GSH and GSSG whose principal components are MEHP and MECPP; and MiBP and MnBP respectively.

To the best of our knowledge, this is the first study to investigate associations between selected oxidative stress biomarkers in adipose tissue and the mixture of blood phthalate metabolites. Other studies have found associations between other biomarkers of oxidative stress related to fertility, such as 8-iso-prostaglandin-F2alpha and its metabolites and urinary phthalate metabolites mixture. In these cases, the phthalate mixture was associated with increased levels of biomarkers of oxidative stress.^68,72^

Phthalates have been suggested to increase oxidative stress in adipose tissue through activation of proliferator-activated receptors γ (PPARγ).^73,74^ These are nuclear receptors expressed in the liver that are involved in fatty acid oxidation, body fat accumulation,^75,76^ as well as in adipogenesis.^74^ In fact, phthalates are considered to be obesogenic molecules, i.e., they have the ability to increase the amount of lipids that accumulate in adipose tissue not only at critical stages of development but also later in life.^77^ This is an important point to consider, as GraMo is an adult cohort and, although exposure might be more relevant at key developmental stages such as pregnancy and infancy,^1,78^ long-term adult exposure to low doses of environmental pollutants should also be considered.

Increased adipogenesis is also relevant to the effects of oxidative stress. Both human and animal studies have shown that this tissue in obese individuals may represent a major source of ROS that plays an important role in the pathogenesis of obesity-associated metabolic syndrome.^36,37^ Furthermore, these ROS are released into the peripheral blood and affect the activities of other organs^36^ and can lead to cardiovascular diseases, diabetes, or cancer, among other health disorders.^1,38−41^

Our study has certain limitations. The cross-sectional design hampers the assumption of causal effects, as reverse causality is possible (although it is not likely biologically plausible). Furthermore, the hospital-based population limits the external validity, although there is no strong reason to consider that our observations are not reproducible at the general population level. Our sample size is also relatively limited, although sufficient to yield several robust and suggestive associations that warrant further confirmation in future studies. In addition, although we used the covariates most commonly used in the literature for fitting models, we did not account for the potential confounding effect of using cosmetics or consuming packaged food, ultraprocesed food, or physical activity because these data were not collected in the original surveys. Another limitation is the use of point samples for estimating phthalate exposure and oxidative stress levels, both of them with potentially high variability due to the instability of the biomarkers. In this cross-sectional study, we posit that serum represents one of the biological matrices most closely aligned with the target matrix, which is adipose tissue. Furthermore, we assume that lifestyle patterns remain relatively constant at the population level. However, we cannot ignore the potential for nondifferential bias arising from variability in serum concentrations.^79,80^

Although there are no significant differences between the main cohort and the subsample analyzed, potential errors in the measurement of the effects of interest must also be considered. In addition, oxidative stress biomarkers were measured years after sample collection; however, it has been shown that proper storage can provide feasible results in relation to the measurement of antioxidant markers in other biological matrices.^81,82^ It is also possible that certain oxidative stress markers not measured in this study and of relevance are related to phthalates, and further studies in this field would be interesting.

Our study also has several strengths. First, we analyzed a large number of phthalate metabolites measured in blood serum, both as individual and combined exposures. Measurement of phthalate metabolites is commonly performed in urine samples as it has some advantages over blood analyses, i.e., higher concentrations of metabolites and lower risk of contamination by the parent compounds.^83^ However, studies in the general population have shown moderate to strong correlations between phthalate metabolite concentrations in urine and serum.^84,85^ Second, despite urine phthalate concentrations and detection rates are frequently higher than those in serum, the latter is closer to the effective dose and site of action so that we are closer to the effective dose.^85,86^ Lastly, biomarkers of oxidative stress were measured in adipose tissue, a highly novel biological matrix in regards to redox assessment, and whose homeostasis is closely linked to obesity-related chronic diseases, such as diabetes and cancer.^36,37^

Thus, our study provides novel insights into the relationship between phthalate exposure redox (un)balance and opens the door to future research to confirm the associations found as well as their long-term health implication.

## Supporting Information Available

List of phthalate metabolites measured in a subsample of the GraMo cohort with their limit of detection and detection rate; phthalate metabolite and oxidative stress biomarker levels of the adult subsample of the GraMo cohort according to the sum of orders of phthalate metabolite levels in tertiles; associations between phthalates and oxidative stress biomarkers; and weighted quantile-sum regression analysis (PDF)
