# Supplementary material for: Serum Phthalate Concentrations and Biomarkers of Oxidative Stress in Adipose Tissue in a Spanish Adult Cohort
Source: Environ Sci Technol. 2024 Apr 23;58(18):7719–30. doi: 10.1021/acs.est.3c07150 (PMC11080070; doi:10.1021/acs.est.3c07150)
Supplement: Supplementary file 1 — es3c07150_si_001.pdf [file es3c07150_si_001.pdf]

Supporting Information

Serum phthalate concentrations and biomarkers of oxidative stress in adipose tissue in a Spanish adult cohort

Celia Pérez-Díaz, Francisco M. Pérez-Carrascosa, Blanca Riquelme-Gallego, Elena Villegas-Arana, Alejandro Joaquin Armendariz, Javier Galindo-Ángel, Hanne Frederiksen, Josefa León, Pilar Requena, Juan Pedro Arrebola

Summary: 31 pages. Table 1. Baseline main characteristics of the subsample adults from the GraMo cohort according to phthalate metabolite sum levels in tertiles. Figure 1. Selection criteria for GraMo cohort participants and flow chart of participants selected for the study subsample. Figure 2. Violin plot of the distribution of participants' BMI in kg/m2 and age in years. Figure 3. Serum concentrations of phthalate metabolites in the study population. Figure 4. Adipose tissue concentrations of oxidative stress biomarkers in the study population. Figure 5. Associations between phthalate metabolites and oxidative stress biomarkers. Figure 6. Estimation of the mixture associations of phthalate metabolites with different immuno-inflammatory biomarker levels. Weighted quantile sum regression (WQS) models.

Supplementary Table S1. Phthalate metabolites measured in a subsample of GraMo cohort (n=143)		
**Human serum metabolite**	**Limit of detection (ng/mL)**	**Detection rate**
Mono-methyl phthalate (MMP)	0.44	60.1
Mono-ethyl phthalate (MEP)	0.65	93.7
Mono-(4-oxopentyl) phthalate (MiPrP)	0.40	0
Mono-propyl phthalate (MPrP)	0.23	0
Mono-iso-butyl phthalate (MiBP)	0.75	71.3
Mono-n-butyl phthalate (MnBP)	0.61	76.9
Mono-(3-hydroxybutyl) phthalate (MHBP)	0.22	5.6
Mono-n-pentyl phthalate (MnPeP)	0.27	0
Mono-(4-hydroxypentyl) phthalate (MHPeP)	0.38	0
Mono-benzyl phthalate (MBzP)	0.26	23.1
Mono-(2-ethyl-hexyl) phthalate (MEHP)	0.74	93.7
Mono-(2-ethyl-5-hydroxyhexyl) phthalate (MEHHP)	0.59	3.5
Mono- (2-ethyl-5-oxohexyl) phthalate (MEOHP)	0.45	8.4
Mono-(2-ethyl-5-carboxypentyl) phthalate (MECPP)	0.25	88.1
Mono-(2-carboxymethyl-hexyl) phthalate (MCMHP)	0.39	75.5
Mono-n-hexyl phthalate (MnHxP)	0.38	0
Mono-(5-hydroxyhexyl) phthalate (MHHxP)	0.26	0

Mono-(5-carboxypentyl) phthalate (MCPeP)	0.20	2.8
Mono-cyclohexyl phthalate (MCHP)	0.27	1.4
Mono-n-heptyl phthalate (MnHPeP)	0.38	18.9
Mono-(6-hydroxyheptyl) phthalate (MHHpP)	0.15	0
Mono-(6-carboxyhexyl) phthalate (MCHxP)	0.23	0.7
Mono-octyl phthalate (MOP)	0.7	0
Mono-3-carboxypropyl phthalate (MCPP)	0.19	0
Mono-iso-nonyl phthalate (MiNP)	0.53	82.5
Mono-hydroxy-iso-nonyl phthalate (MHiNP)	0.4	0
Mono-oxo-iso-nonyl phthalate (MOiNP)	0.31	0
Mono-carboxy-iso-octyl phthalate (MCiOP)	0.13	12.6
Mono-iso-decyl phthalate (MiDP)	0.72	46.2
Mono-(9-hydroxydecyl) phthalate (MHiDP)	0.31	0
Mono-(9-oxodecyl) phthalate (MOiDP)	0.31	0
Mono-(9-carboxynonyl) phthalate (MCiDP)	0.32	0

**Supplementary Table S2. Phthalate metabolite and oxidative stress biomarker levels of the adult subsample of the GraMo cohort according to the sum of orders of phthalate metabolite levels in tertiles (n = 143)**			
**Characteristics**	**Phthalate metabolite sum**		
	**1st tertile (lowest levels)**	**2nd tertile**	**3rd tertile (higher levels)**
**n (%)**	55 (38.5%)	50 (35%)	38 (26.6%)
**Biomarkers of oxidative stress [median (SD)]**			
GPx	16.68 (14.00)	14.77 (13.42)	16.18 (12.44)
GRd	0.17 (0.18)	0.40 (1.66)	0.14 (0.11)
GST	43.23 (104.46)	28.16 (32.86)	33.06 (34.18)
GSH	14.90 (46.68)	8.05 (10.57)	12.22 (17.58)
GSSG	28.21 (64.45)	19.26 (26.88)	20.84 (22.18)
GSH/GSSG	1.40 (4.12)	0.91 (1.80)	1.35 (3.08)
HO-1	28.30 (35.43)	22.92 (19.68)	19.29 (24.75)
SOD	22.89 (32.69)	30.05 (43.84)	20.54 (32.97)
TBARS	7.66 (8.15)	6.09 (6.71)	7.28 (6.86)
8OHdG	4.47 (11.13)	7.54 (33.29)	4.02 (9.09)
**Phtalate metabolite serum levels [median (SD)]**			
MMP (ng/mL)	5.29 (7.18)	4.42 (5.93)	5.52 (5.68)

MEP (ng/mL)	13.08 (29.59)	19.97 (23.41)	30.77 (21.36)
MiBP (ng/mL)	1.02 (1.88)	8.55 (13.39)	15.25 (17.32)
MnBP (ng/mL)	0.86 (1.84)	7.62 (12.43)	16.64 (18.60)
MBzP (ng/mL)	0.15 (0.10)	0.24 (0.32)	0.55 (0.50)
MEHP (ng/mL)	1.54 (1.39)	4.15 (4.90)	3.12 (3.12)
MECPP (ng/mL)	0.76 (1.09)	1.44 (2.01)	1.74 (0.79)
MCMHP (ng/mL)	1.05 (1.61)	1.57 (2.16)	2.37 (1.52)
MiNP (ng/mL)	0.93 (1.11)	1.85 (1.43)	1.87 (1.09)
MiDP (ng/mL)	1.60 (7.77)	1.24 (1.22)	1.84 (1.89)

Abbreviations: GPx: glutathione peroxidase; GRd: glutathione reductase; GST: total glutathione; GSH: reduced glutathione; GSSG: oxidised glutathione; HO-1: hemeoxygenase-1; SOD: superoxide dismutase; TBARS: thiobarbituric acid reactive substances; 8OHdG: 8-hydroxy-deoxyguanosine; MMP: Mono-methyl phthalate; MEP: mono-ethyl phthalate; MiBP: mono-iso-butyl phthalate; MnBP: mono-n-butyl phthalate; MBzP: mono-benzyl phthalate; MEHP: mono-(2-ethyl-hexyl) phthalate; MECPP: mono-(2-ethyl-5-carboxypentyl) phthalate; MCMHP mono-(2-carboxymethyl-hexyl) phthalate; MiNP: mono-iso-nonyl phthalate; MiDP: mono-isodecyl phthalate

**Supplementary Table S3**. **Associations between phthalates and oxidative stress biomarkers.**												
		MMP	MBzP	MEP	MiBP	MnBP	MEHP	MECPP	MCMHP	MiNP	MiDP	Phthalate sum
GPx	**Model 1**	**0.36 (0.11, 0.62)**	3.09 (-0.89, 7.08)	0.02 (-0.04, 0.09)	**0.28 (0.15, 0.41)**	**0.20 (0.07, 0.33)**	-0.26 (-0.72, 0.20)	-0.06 (-1.13, 1.01)	0.28 (-0.62, 1.17)	**-2.07 (-3.32, -0.83)**	-1.96 (-5.44, 1.52)	-0.08 (-0.56, 0.41)
	**Model 2**	**0.46 (0.18, 0.75)**	3.79 (-0.40, 7.98)	0.03 (-0.04, 0.09)	**0.28 (0.14, 0.43)**	**0.22 (0.08, 0.37)**	-0.31 (-0.81, 0.18)	-0.18 (-1.36, 1.01)	0.24 (-0.76, 1.23)	**-2.26 (-3.67, -0.85)**	-1.95 (-5.71, 1.81)	-0.08 (-0.61, 0.45)
GRd	**Model 1**	**0.00 (0.00, 0.01)**	-0.01 (-0.06, 0.04)	0.00 (0.00, 0.00)	0.00 (0.00, 0.00)	0.00 (0.00, 0.00)	0.00 (-0.01, 0.00)	0.01 (0.00, 0.02)	0.00 (-0.01, 0.01)	-0.01 (-0.03, 0.01)	0.00 (-0.04, 0.05)	0.00 (-0.01, 0.00)
	**Model 2**	**0.00 (0.00, 0.01)**	-0.01 (-0.06, 0.04)	0.00 (0.00, 0.00)	0.00 (0.00, 0.00)	0.00 (0.00, 0.00)	0.00 (-0.01, 0.00)	0.01 (-0.01, 0.02)	0.00 (-0.01, 0.01)	-0.01 (-0.03, 0.01)	0.01 (-0.04, 0.05)	0.00, (-0.01, 0.01)
GST	**Model 1**	-0.12 (-0.77, 0.54)	-1.47 (-11.00, 8.06)	-0.05 (-0.21, 0.10)	-0.09 (-0.43, 0.25)	-0.12 (-0.44, 0.20)	0.39 (-0.70, 1.47)	0.73 (-1.89, 3.35)	0.34 (-1.80, 2.48)	-1.47 (-4.65, 1.72)	-1.34 (-9.71, 7.03)	-0.11 (-1.28, 1.07)
	**Model 2**	-0.20 (-0.90, 0.50)	-2.66 (-12.62, 7.29)	-0.05 (-0.21, 0.11)	-0.15 (-0.49, 0.19)	-0.19 (-0.52, 0.15)	0.35 (-0.81, 1.50)	0.76 (-1.95, 3.47)	0.16 (-2.12, 2.44)	-2.15 (-5.36, 1.06)	-0.93 (-9.61, 7.75)	-0.20 (-1.40, 1.00)
GSSG	**Model 1**	-0.17 (-0.44, 0.10)	1.93 (-2.02, 5.88)	-0.01 (-0.07, 0.06)	0.02 (-0.12, 0.16)	0.02 (-0.11, 0.15)	0.17 (-0.27, 0.62)	0.91 (-0.17, 1.98)	0.02 (-0.88, 0.92)	0.06 (-1.29, 1.41)	**3.65 (0.39, 6.90)**	0.17 (-0.32, 0.65)
	**Model 2**	-0.19 (-0.49, 0.11)	1.81 (-2.28, 5.89)	0.00 (-0.07, 0.06)	0.03 (-0.11, 0.17)	0.03 (-0.11, 0.17)	0.15 (-0.32, 0.62)	0.98 (-0.16, 2.11)	0.10 (-0.84, 1.04)	0.16 (-1.26, 1.58)	**4.03 (0.51, 7.56)**	0.17 (-0.34, 0.67)
GSH	**Model 1**	0.12 (-0.30, 0.53)	-1.15 (-7.45, 5.14)	-0.02 (-0.12, 0.08)	-0.09 (-0.31, 0.13)	-0.10 (-0.31, 0.11)	0.09 (-0.63, 0.80)	-0.32 (-1.99, 1.35)	-0.05 (-1.44, 1.34)	-1.61 (-3.61, 0.38)	-2.90 (-8.39, 2.59)	-0.20 (-0.96, 0.56)
	**Model 2**	0.07 (-0.39, 0.53)	-1.83 (-8.17, 4.50)	-0.01 (-0.11, 0.10)	-0.12 (-0.35, 0.11)	-0.15 (-0.38, 0.07)	0.05 (-0.69, 0.79)	-0.59 (-2.36, 1.19)	-0.49 (-1.93, 0.96)	-2.00 (-4.23, 0.22)	-2.40 (-8.19, 3.40)	-0.26 (-1.05, 0.53)
GSSG/GSH	**Model 1**	**-0.02(-0.04, 0.00)**	0.12 (-0.17, 0.40)	0.00 (0.00, 0.01)	0.00 (-0.01, 0.01)	0.00 (-0.01, 0.01)	0.02 (-0.01, 0.05)	**0.12 (0.04, 0.19)**	0.02 (-0.05, 0.09)	0.04 (-0.06, 0.14)	0.23 (-0.02, 0.49)	0.01 (-0.02, 0.05)
	**Model 2**	**-0.02 (-0.04, 0.00)**	0.16 (-0.15, 0.48)	0.00 (0.00, 0.01)	0.00 (-0.01, 0.01)	0.00 (-0.01, 0.01)	0.02 (-0.02, 0.05)	**0.13 (0.04, 0.21)**	0.02 (-0.05, 0.09)	0.05 (-0.07, 0.16)	0.29 (0.00, 0.57)	0.02 (-0.02, 0.06)
HO-1	**Model 1**	0.27 (-0.12, 0.67)	-4.29 (-10.14, 1.56)	0.00 (-0.10, 0.09)	0.03 (-0.18, 0.23)	-0.07 (-0.27, 0.12)	-0.07 (-0.74, 0.60)	-0.15 (-1.76, 1.47)	1.36 (-0.02, 2.75)	-0.49 (-2.47, 1.50)	-0.15 (-5.34, 5.03)	-0.37 (-1.11, 0.37)
	**Model 2**	0.38 (-0.02, 0.79)	-4.14 (-10.22, 1.95)	-0.01 (-0.11, 0.09)	0.04 (-0.17, 0.25)	-0.07 (-0.28, 0.14)	-0.10 (-0.78, 0.58)	-0.82 (-2.42, 0.77)	0.91 (-0.50, 2.33)	-0.78 (-2.73, 1.29)	1.44 (-3.78, 6.66)	-0.50 (-1.23, 0.23)
SOD	**Model 1**	0.01 (-0.46, 0.48)	-4.66 (-11.92, 2.60)	-0.04 (-0.15, 0.07)	0.08 (-0.15, 0.32)	0.06 (-0.17, 0.30)	0.67 (-0.13, 1.47)	-0.70 (-2.66, 1.26)	-0.43 (-1.98, 1.12)	**-2.51 (-4.98, -0.05)**	-3.04 (9.23, 3.16)	-0.45, (-1.31, 0.41)
	**Model 2**	0.04 (-0.48, 0.57)	-4.13 (-11.61, 3.35)	-0.03 (-0.15, 0.08)	0.11 (-0.15, 0.37)	0.09 (-0.16, 0.34)	0.73 (-0.11, 1.57)	-1.08 (-3.21, 1.04)	-0.92 (-2.63, 0.78)	-2.60 (-4.31, 0.11)	-2.72 (-9.22, 3.78)	-0.56 (-1.45, 0.33)

TBARS	**Model 1**	**0.19 (0.04, 0.34)**	1.61 (-0.83, 4.04)	0.01 (-0.03, 0.05)	**0.12 (0.04, 0.19)**	**0.08 (0.00, 0.16)**	-0.01 (-0.28, 0.26)	-0.07 (-0.73, 0.58)	0.05 (-0.48, 0.58)	**-1.28 (-2.04, -0.52)**	-0.99 (-3.08, 1.11)	0.03 (-0.26, 0.33)
	**Model 2**	**0.23 (0.06, 0.40)**	1.74 (-0.72, 4.19)	0.02 (-0.02, 0.06)	**0.13 (0.05, 0.21)**	**0.10 (0.02, 0.19)**	-0.04 (-0.33, 0.25)	-0.12 (-0.81, 0.56)	0.00 (-0.57, 0.57)	**-1.49 (-2.32, -0.66)**	-0.89 (-3.13, 1.35)	0.04 (-0.26, 0.35)
8OHdG	**Model 1**	-0.03 (-0.08, 0.01)	-0.52 (-1.17, 0.13)	0.00 (-0.01, 0.01)	**-0.03 (-0.06, 0.00)**	-0.02 (-0.04, 0.01)	0.00 (-0.07, 0.07)	-0.07 (-0.26, 0.12)	-0.04 (-0.20, 0.11)	-0.03 (-0.27, 0.20)	-0.04 (-0.65, 0.56)	-0.05 (-0.12, 0.03)
	**Model 2**	-0.04 (-0.10, 0.02)	-0.48 (-1.16, 0.21)	0.00 (-0.02, 0.01)	**-0.03 (-0.06, 0.00)**	-0.01 (-0.04, 0.01)	0.00 (-0.08, 0.09)	-0.07 (-0.28, 0.13)	-0.05 (-0.22, 0.12)	-0.05 (-0.33, 0.23)	-0.06 (-0.78, 0.66)	-0.05 (-0.14, 0.04)

Cells display the changes in folds of oxidative stress biomarker concentration due to an increase in the interquartile range of phthalate metabolite level or a change in category for categorized variables, with 95% confidence intervals in parentheses. Bold text indicates that the confidence interval does not include the value of "1" (statistical significance). Phthalate metabolites were logarithmically transformed.

Model 1 is adjusted for sex, age, body mass index, occupation, alcohol consumption, smoking and hospital where surgery was performed. Model 2, in addition to these variables, was adjusted for vegetable food consumption.

*MiDP and MBzP were categorized into two categories as described in the methods section.

GPx, glutathione peroxidase; GRd, glutathione reductase; GST, total glutathione, GSH, reduced glutathione, GSSG, oxidised glutathione; GSSG/GSH, oxidised glutathione/reduced glutathione ratio; HO-1, hemeoxygenase-1; SOD, superoxide dismutase; TBARS, thiobarbituric acid reactive substances; 8OHdG, 8-hydroxy-deoxyguanosine; MMP, Mono-methyl phthalate; MBzP, mono-benzyl phthalate; MEP, mono-ethyl phthalate; MiBP, mono-iso-butyl phthalate; MnBP, mono-n-butyl phthalate; MEHP, mono-(2-ethyl-hexyl) phthalate; MECPP, mono-(2-ethyl-5- carboxypentyl) phthalate; MCMHP mono-(2-carboxymethyl-hexyl) phthalate; MiNP, mono-iso-nonyl phthalate; MiDP, mono-isodecyl phthalate.

**Supplementary Table S4. Weighted Quantile-sum Regression analysis.**						
	Positive mixture effect			Negative mixture effect		
	β	Standard error	P-value	β	Standard error	P-value
GPx	0,682	2,739	0,804	-0,993	3,626	0,785
GRd	0,212	0,305	0,489	0,094	0,330	0,775
GST	-29,292	17,773	0,103	-39,967	21,117	0,062
GSSG	-10,267	8,049	0,206	-19,591	8,893	0,030*
GSH	-24,944	12,040	0,042*	-30,089	13,162	0,025*
GSSG/GSH	-0,533	0,974	0,586	0,240	0,874	0,784
HO-1	-6,273	5,353	0,245	-7,840	7,065	0,270
SOD	-2,391	10,118	0,814	-11,670	10,763	0,280
TBARS	1,083	1,780	0,545	-3,042	2,042	0,140
8OHdG	-1,826	2,830	0,521	-0,231	2,721	0,933

*Statistically significant

Model 1 is adjusted for sex, age, body mass index, occupation, alcohol consumption, smoking and hospital where surgery was performed. Model 2, in addition to these variables, was adjusted for vegetable food consumption.

GPx, glutathione peroxidase; GRd, glutathione reductase; GST, total glutathione, GSH, reduced glutathione, GSSG, oxidised glutathione; GSSG/GSH, oxidised glutathione/reduced glutathione ratio; HO-1, hemeoxygenase-1; SOD, superoxide dismutase; TBARS, thiobarbituric acid reactive substances; 8OHdG, 8-hydroxy-deoxyguanosine; MMP, Mono-methyl phthalate; MBzP, mono-benzyl phthalate; MEP, mono-ethyl phthalate; MiBP, mono-iso-butyl phthalate; MnBP, mono-n-butyl phthalate; MEHP, mono-(2-ethyl-hexyl) phthalate; MECPP, mono-(2-ethyl-5- carboxypentyl) phthalate; MCMHP mono-(2-carboxymethyl-hexyl) phthalate; MiNP, mono-iso-nonyl phthalate; MiDP, mono-isodecyl phthalate.
